# Metric System in Prescriptions

**Published:** 1876-06

**Authors:** 


OUR Senior Editor has been absent for some time, on a visit to Virginia and the Centennial. This will account for the meagreness of editorial matter in the present issue. He will probably give some account of his observations in our next number.

*Metric System in Prescriptions*—A correspondent of the *Medical Record*, New York, urges the importance of adopting the metric system in our prescriptions. He is right, and we do respectfully propose to all our cotemporaries to take at once a decided stand in favor of this proposition. Let us urge the American Medical Association, soon to assemble in Philadelphia, to take action upon the subject. Our present system is exceedingly imperfect and *unsafe.* The ounce ( ℥ ) mark and the drachm (ʒ) mark are too much alike, and lead to many and serious mistakes. There is also want of uniformity in that we have three different quantities represented by the ounce mark. For instance, ℥ i av.=437.5 grs., f ℥ i=455.56 grs., and ℥ i (Troy) =480 grs.

In the metric system different characters are not required, every quantity of each ingredient being expressed in fractions, or multiples of a common unit, which is a gramme—thus:

| | | Grammes. |
|---|---|---|
| R. | Hydrarg. proto. iod........ ....................... | 1 |
| | Ext. lactucarii................. ..................... | 3 |
| | M. Ft. pil. No. LX. | |
| R. | Strychniai sulph................................... | Grammes. |
| | Acidi arseniosi aa................. ................. | 0.03 |
| | Quinae sulph........ ................................ | 2 |
| | Ext. gentian com.......... ...................... ...... | 9.5 |
| | M. Ft. pil. XXX. | |

The following table for reducing apothecaries weights to the metric system, we extract from the *Boston Journal of Chemistry:*

| Apothecaries Weights. | Grammes | Apothecaries' Weights. | Grammes |
|---|---|---|---|
| Grain $\frac{1}{10}$ | 0.006 | Grains 80 | 5.18 |
| " $\frac{1}{8}$ | 0.008 | " 90 | 5.83 |
| " $\frac{1}{6}$ | 0.011 | " 96 | 6.22 |
| " $\frac{1}{5}$ | 0.012 | " 100 | 6.48 |
| " $\frac{1}{4}$ | 0.016 | " 120 (dr.ij) | 7.77 |
| " $\frac{1}{3}$ | 0.021 | " 150 | 9.72 |
| " $\frac{1}{2}$ | 0.032 | " 160 | 10.37 |
| " $\frac{2}{3}$ | 0.042 | " 180 (dr.iij) | 11.66 |
| " $\frac{3}{4}$ | 0.048 | " 200 | 12.96 |
| " 1 | 0.065 | " 240 (oz.ss) | 15.55 |
| Grains 2 | 0.13 | Drachms 5 | 19.44 |
| " 3 | 0.194 | " $5\frac{1}{2}$ | 21.38 |
| " 4 | 0.259 | " 6 | 23.33 |
| " 5 | 0.324 | " 7 | 27.21 |
| " 6 | 0.389 | " 8 (oz.j) | 31.1 |
| " 7 | 0.453 | " 9 | 34.99 |
| " 8 | 0.518 | " 10 | 38.88 |
| " 9 | 0.583 | " 12 | 46.66 |
| " 10 | 0.648 | " 14 | 54.35 |
| " 12 | 0.777 | " 16 (oz.ij) | 62.2 |
| " 14 | 0.907 | " 18 | 69.99 |
| " 15 | 0,972 | " 20 | 77.76 |
| " 16 | 1.037 | " 24 (oz.iij) | 93.31 |
| " 18 | 1.166 | Ounces $3\frac{1}{2}$ | 108.81 |
| " 20 (scr.j) | 1.296 | " 4 | 124.41 |
| " 24 | 1.555 | " $4\frac{1}{2}$ | 139.91 |
| " 30 | 1.944 | " 5 | 155.51 |
| " 32 | 2.074 | " $5\frac{1}{2}$ | 171. |
| " 36 | 2.333 | " 6 | 186.62 |
| " 40 (scr.ij) | 2.592 | " $6\frac{1}{2}$ | 202.12 |
| " 48 | 3.11 | " 7 | 217.72 |
| " 50 | 3.24 | " 8 | 248.82 |
| " 60 (gr.j) | 3.89 | " 9 | 279.92 |
| " 72 | 4.66 | " 10 | 311.03 |

